# Incidence of Cancer in ANCA-Associated Vasculitis: A Meta-Analysis of Observational Studies

**DOI:** 10.1371/journal.pone.0126016

**Published:** 2015-05-14

**Authors:** Weifeng Shang, Yong Ning, Xiu Xu, Menglan Li, Shuiming Guo, Min Han, Rui Zeng, Shuwang Ge, Gang Xu

**Affiliations:** Department of Nephrology, Tongji hospital affiliated to Tongji medical college, Huazhong University of Science and Technology, Wuhan, Hubei, China; Nippon Medical School Graduate School of Medicine, JAPAN

## Abstract

**Objective:**

The purpose of this paper is to examine cancer incidence in patients with ANCA-associated vasculitis (AASV) derived from population-based cohort studies by means of meta-analysis.

**Methods:**

Relevant electronic databases were searched for studies characterizing the associated risk of overall malignancy in patients with AASV. Standardized incidence rates (SIRs) with 95% confidence intervals (CIs) were used to evaluate the strength of association. We tested for publication bias and heterogeneity and stratified for site-specific cancers.

**Results:**

Six studies (n = 2,578) were eventually identified, of which six provided the SIR for overall malignancy, five reported the SIR for non-melanoma skin cancer (NMSC), four for leukemia, five for bladder cancer, three for lymphoma, three for liver cancer, four for lung cancer, three for kidney cancer, four for prostate cancer, four for colon cancer and four for breast cancer. Overall, the pooled SIR of cancer in AASV patients was 1.74 (95%CI = 1.37–2.21), with moderate heterogeneity among these studies (I^2^ = 65.8%, P = 0.012). In sub-analyses for site-specific cancers, NMSC, leukemia and bladder cancer were more frequently observed in patients with AASV with SIR of 5.18 (95%CI = 3.47–7.73), 4.89 (95%CI = 2.93–8.16) and 3.84 (95%CI = 2.72–5.42) respectively. There was no significant increase in the risk of kidney cancer (SIR = 2.12, 95%CI = 0.66–6.85), prostate cancer (SIR = 1.45, 95%CI = 0.87–2.42), colon cancer (SIR = 1.26, 95%CI = 0.70–2.27), and breast cancer (SIR = 0.95, 95%CI = 0.50–1.79). Among these site-specific cancers, only NMSC showed moderate heterogeneity (I^2^ = 55.8%, P = 0.06). No publication bias was found by using the Begg’s test and Egger's test.

**Conclusions:**

This meta-analysis shows that AASV patients treatment with cyclophosphamide (CYC) are at increased risk of late-occurring malignancies, particularly of the NMSC, leukemia and bladder cancer. However, there is no significant association between AASV and kidney cancer, prostate cancer, colon cancer and breast cancer. These findings emphasize monitoring and preventative management in AASV patients after cessation of CYC therapy is momentous.

## Introduction

The anti-neutrophil cytoplasm antibody associated vasculitides (AASVs), including graulomatosis with polyangiitis (GPA, Wegener’s granulomatosis) and microscopic polyangiitis (MPA), are a group of multisystem disorders characterized by necrotizing inflammation of small blood vessels[1]. Although, AASV involves small vessels with predilection for the kidneys, lungs, and peripheral nervous system in most of the patients, any organ system can be affected. The disease usually presents in the middle aged or elderly, with a peak incidence of 65 per million/year in those aged 65–74 years[2]. There is a reported mortality of 80% at 1 year in untreated patients[2]. Since the late 1970s, the introduction of glucocorticoids and cyclophosphamide (CYC) as standard treatments has improved the prognosis considerably[3]. An analysis of four multicenter trials reported cumulative survival at 1, 2 and 5 years of 88%, 85% and 78%, respectively[4]. With standard therapy regimen, remission can be induced in about 70–90% of patients[5]. Nevertheless, 25–50% of the patients will relapse[6], leading to increased duration and quantity of CYC which would cause toxic effects especially for cardiovascular and cancer morbidity[7]. The latter has drawn more attention recently. Much effort has been devoted to this field which demonstrated the SIRs for overall cancer has increased to more than 1.6 associated with AASV[6,8–13], but the risk of overall cancer appeared to be somewhat reduced in one study (SIR = 0.8, 95%CI = 0.5–1.4)[14]. Moreover, cancer type-specific analyses demonstrated an significantly increased risk of non-melanoma skin cancer (NMSC)[8–12], leukemia[9–12], and bladder cancer[6,9–12,15] in some studies. However, the reported risk is different. Recently, the comprehensive review performed by Mahr et al.[16] has shown the current understanding of the potential link between AASV and the occurrence of cancer, but no meta-analysis has been used to examine the relationship. Given the fact that individual studies may have insufficient statistical power because of sample size, therefore, we undertook the present meta-analysis to quantitatively confirm the incidence of cancer in AASV patients versus the general population, which may provide a realistic perspective on risk in the clinical setting.

## Materials and Methods

### Search strategy and study selection

A PubMed and EMBASE databases were searched systematically for all articles published before September 1st, 2014. The search terms used were “cancer and ANCA-associated vasculitis”,“cancer and AASV”,“cancer and WG”,“cancer and GPA”,“cancer and MPA”, “epidemiology and ANCA-associated vasculitis”. Two investigators (WS and SG), using these parameters, independently filtered out all the eligible articles and hand-searched references of retrieved papers for additional available studies. We did not include unpublished or abstract only publications. No restriction was placed on language. Conflicting results were resolved by consensus and endnote was used to merge retrieved citations.

### Inclusion criteria

Included studies met the following criteria: (1) they were cohort studies that estimating the influence of AASV on cancer risk with odds ratio (OR), risk ratio (RR), hazard ratio (HR), or standardized incidence/mortality rate(SIR/SMR) and their 95% confidence intervals (CIs) of overall cancer. (2) defined AASV as one of the exposure interests and cancer as one of the outcome of interests.

### Exclusion criteria

The exclusion criteria included: reviews, case reports, conference publications, cross-sectional studies, studies limiting the population age, studies not specifying the types of tumor, and studies focusing on demonstrate incidence of cancer before the diagnosis of AASV. If a cohort study was reported in more than one publication, we chose the largest sample size or the latest article.

### Data extraction and Quality evaluation

The following data were extracted independently by two investigators (WS and SG) from the included studies: the authors, publication year, country, study design, AASV phenotypes studied, period of follow-up, mean/median observation period, cumulative observation period, number of patients studied, number of cancers observed in the cohort, patients’ gender and age, and SIR with its 95% CI, gender of cancer patients, main therapy. When needed, we contacted the original author for clarification. This study complied with meta-analysis of observational studies in Epidemiology (S1 MOOSE Checklist)[17]. The quality of each study was independently evaluated by two investigators (WS and SG) using the Newcastle-Ottawa Scale (NOS)[18]. The NOS, including selection, comparability and outcome, is a scale for assessing the quality of published non-randomized studies. The study which met at least five NOS criteria was considered to be a high quality study. Discrepancies between investigators were solved by consensus.

### Data Synthesis and Analysis

In this meta-analysis, we collected SIR with 95%CI to combine the data and assessed heterogeneity of the mean difference with the Chi-squared based Q-statistic test. If the P value of the heterogeneity Q-statistic was less than 0.10, the random-effects model was used to calculate the pooled SIRs[19]. On the contrary, the fixed-effects model was selected[20]. We also quantified the effect of heterogeneity using the I^2^ index[21]. I^2^ values of 25%, 50% and 75% indicate low, moderate and severe statistical heterogeneity, respectively. Publication bias was evaluated using the Begg’s test and Egger’s test[22,23]. P value <0.10 was considered significant. All analyses were performed in Stata 10.0 (College Station, TX, USA).

## Results

### Studies included in the meta-analysis

**Fig 1:**
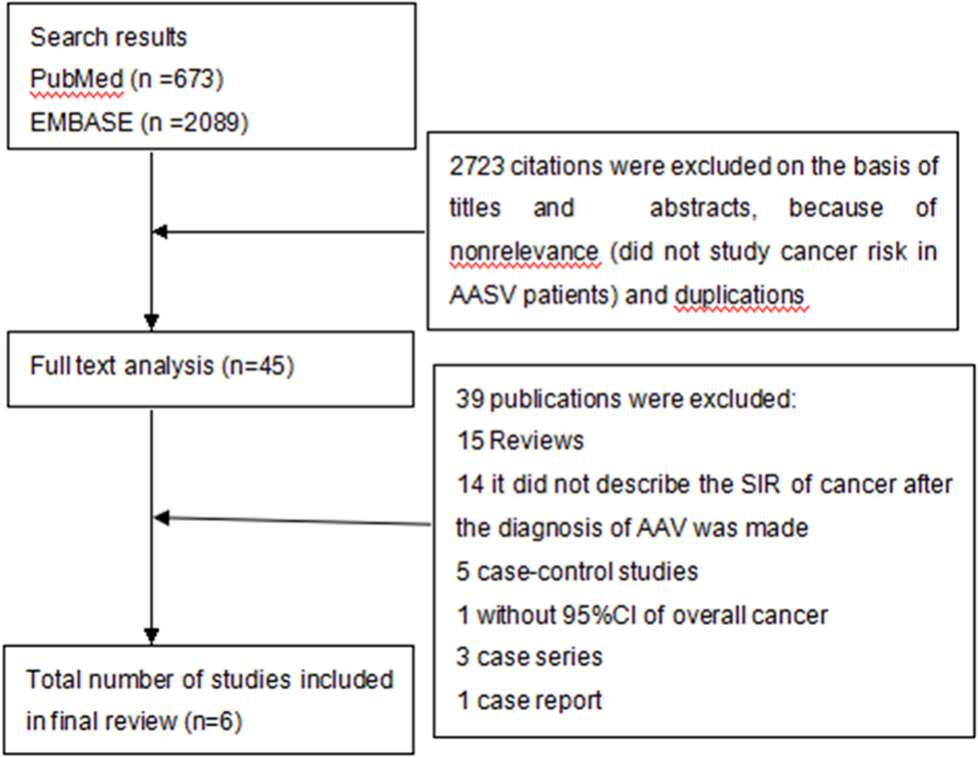
Literature search flow diagram.

As shown in Fig 1, our initial search rendered 2,762 potentially relevant articles, from which six cohort studies were included[8–12,14]. Three studies were monocentric cohorts[8,12,14], two study nationwide registry linkage[9,10], and one was a multinational study[11]. Among these studies, two were carried out in Sweden, one in Germany, one in Poland, one in Denmark and one in Europe and Mexico. They were published between 1998 and 2013. Mean or median observation period for ascertained cases ranged from 4.58 to 7 years. In this meta-analysis, we are including 258 cases of cancers identified in a total cohort of 2,578 individuals, 1,191 women (46%) and 1,387 men (54%), with a diagnosis of AASV selected characteristics of the included studies are shown in Table 1. While Table 2 shows the detailed ratings of study quality for NOS.

**Table 1:** Characteristics of studies of ANCA-associated vasculitis (AASV) and cancer incidence.

Author(year)	Westman et al. 1998[8]	Knight et al. 2002[9]	Faurschou et al. 2008[10]	Holle et al. 2011[14]	Heijl et al. 2011[11]	Zycinska et al. 2013[12]
Country	Sweden	Sweden	Denmark	Germany	Europe, Mexico	Poland
Study design	Monocenter clinical cohort study	nationwide hospital discharge database study	nationwide hospital discharge database study	monocenter clinical cohort study	multicenter clinical trials	monocenter clinical cohort study
AASV phenotypes studied	GPA /MPA	GPA	GPA	GPA	GPA /MPA	Pulmonary vasculitis
Follow-up period	1971–1993	1969–1994	1973–1999	1966–2005	1995–2007	1990–2008
Mean/median observation period, years	4.58	NR	6	NR	4.95	7
Cumulative observation period, person-year	944	5708	2121	2572	2650	NR
Patients with AASV, n	123	1065	293	445	535	117
Gender, % male	64.2%	53%	53.2%	50.1%	53.8%	67%
Medium/Mean age, years	61.8	NR	59	51.7	57.7	64.5
Cancer(following the Diagnosis of AASV), n	15	110	50	18	50	15
SIR and 95%CI	1.6 (0.9–2.7)	2.0 (1.7–2.5)	2.1 (1.5–2.7)	0.8 (0.5–1.4)	1.6 (1.2–2.1)	2.5 (1.2–2.9)
Gender of cancer patients, % male or SIR	NR	SIR 2.1	NR	SIR 1.12	63.0%	NR
Main therapy	CYC/GC	CYC/GC	CYC/GC	CYC/GC	CYC/GC	CYC/GC

Abbreviations: NR, not reported; SIR, standardized incidence rate; CI,confidence interval; GPA, graulomatosis with polyangiitis; MPA, microscopic polyangiitis; CYC, cyclophosphamide; GC, glucocorticoids.

**Table 2:** Assessment of study quality.

References	Quality indications form of Newcastle-Ottawa Scale									Total stars
	1	2	3	4	5a	5b	6	7	8	
Westman et al. 1998[8]	Yes	Yes	Yes	Yes	Yes	Yes	Yes	Yes	Yes	9
Knight et al. 2002[9]	Yes	Yes	Yes	No	Yes	Yes	Yes	Yes	Yes	8
Faurschou et al. 2008[10]	Yes	Yes	Yes	No	Yes	Yes	Yes	Yes	Yes	8
Holle et al. 2011[14]	Yes	Yes	Yes	No	Yes	Yes	Yes	No	Yes	7
Heijl et al. 2011[11]	Yes	Yes	Yes	Yes	Yes	Yes	Yes	No	Yes	8
Zycinska et al. 2013[12]	Yes	Yes	Yes	No	Yes	Yes	No	No	No	5

For cohort studies: 1, representativeness of exposed cohort; 2, selection of the nonexposed cohort; 3, ascertainment of exposure; 4, outcome of interest not present at start; 5a, cohorts comparable on basis of main factor; 5b, cohorts comparable on any additional factor; 6, assessment of outcome with independency; 7, follow-up long enough for outcomes to occur; 8, complete accounting for cohorts or subjects lost to follow-up unlikely to introduce bias.

### Overall cancer risk in AASV

**Fig 2:**
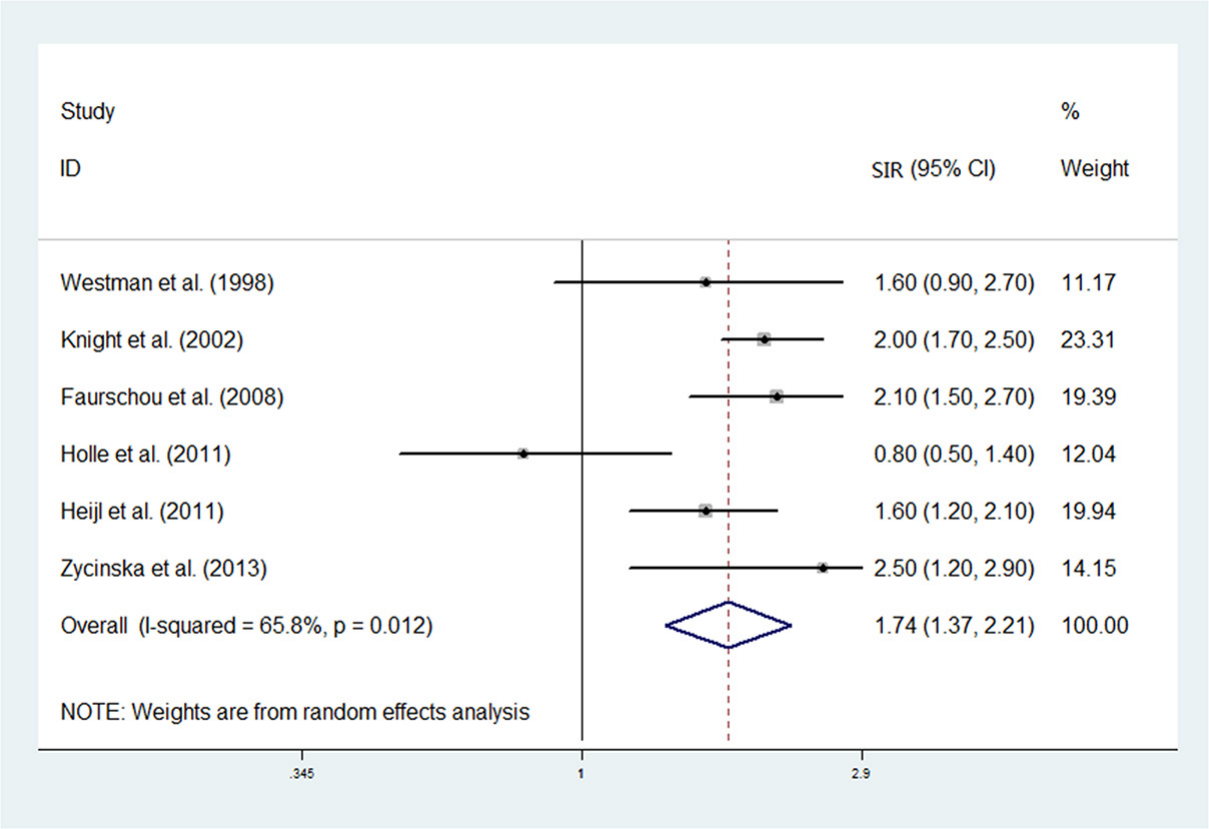
Forest plot of association between ANCA-associated vasculitis (AASV) and overall cancer.

The SIR of cancers within the six individual study populations ranged between 0.8 and 2.5, with an overall meta-analytical SIR of 1.74 (95%CI = 1.37–2.21), and the heterogeneity was moderate (I^2^ = 65.8%, P = 0.012). The forest plot of SIR is shown in Fig 2.

### Organ-specific Cancer Associated with AASV

We performed subgroup analyses for all site specific cancers that have been reported by more than one included study (Table 3). This subgroup analyses did not show evidence of significant heterogeneity (I^2^ = 0) except for the NMSC (I^2^ = 55.8%, P = 0.06). The result revealed that NMSC, leukemia and bladder cancer were more frequently observed in patients with AASV than in the entire population, with SIR of 5.18 (95%CI = 3.47–7.73), 4.89 (95%CI = 2.93–8.16) and 3.84 (95%CI = 2.72–5.42) respectively, followed by lymphoma (SIR = 3.79, 95%CI = 1.87–7.69), liver cancer (SIR = 3.50, 95%CI = 1.45–8.43), lung cancer (SIR = 1.67, 95%CI = 1.07–2.60). However, there was no significant increase in the risk of kidney cancer (SIR = 2.12, 95%CI = 0.66–6.85), prostate cancer (SIR = 1.45, 95%CI = 0.87–2.42), colon cancer (SIR = 1.25, 95%CI = 0.66–2.38), and breast cancer (SIR = 0.95, 95%CI = 0.50–1.79). In organ-specific cancer with only one study, formal meta-analyses were not performed. However, it is still worth noting that the greatly high SIRs for cancers of the testis (45.7)[8], vulva (32.9)[8] and nose and ear (14.1)[9] reported relating to AASV.

**Table 3:** Pooled site-specific cancer risks in patients with ANCA-associated vasculitis (AASV).

Site-specific cancers	References(no.of studies)	Pooled SIR (95% CI)^a^	Heterogeneity	
			**I** ^**2**^ **(%)** ^**b**^	**P value(Q test)** ^**c**^
Non-melanoma skin cancer	Westman et al.(1998), Knight et al.(2002), Faurschou et al.(2008),Heijl et al.(2011), Zycinska et al.(2013). (5)	5.18(3.47–7.73)	55.8%	0.060
Leukemia	Knight et al.(2002), Faurschou et al. (2008), Heijl et al.(2011), Zycinska et al.(2013). (4)	4.89(2.93–8.16)	0%	0.909
Bladder	Westman et al.(1998), Knight et al.(2002), Faurschou et al.(2008),Heijl et al.(2011), Zycinska et al.(2013). (5)	3.84(2.72–5.42)	0%	0.809
Lymphoma	Westman et al.(1998), Knight et al.(2002), Heijl et al.(2011). (3)	3.79(1.87–7.69)	0%	0.639
Liver	Knight et al.(2002), Faurschou et al. (2008), Heijl et al.(2011).(3)	3.50(1.45–8.43)	0%	0.871
Lung	Knight et al.(2002), Faurschou et al.(2008), Heijl et al.(2011), Zycinska et al.(2013). (4)	1.67(1.07–2.60)	0%	0.915
Kidney^e^	Westman et al.(1998), Knight et al.(2002).(2)	2.12(0.66–6.85)	0%	0.711
Prostate	Westman et al.(1998), Knight et al.(2002), Faurschou et al. (2008), Heijl et al.(2011). (4)	1.44(0.88–2.34)	0%	0.395
Colon	Knight et al.(2002), Faurschou et al.(2008), Heijl et al.(2011)^d^, Zycinska et al.(2013). (4)	1.26(0.70–2.27)	0%	0.642
Breast	Westman et al.(1998), Knight et al.(2002), Faurschou et al. (2008), Heijl et al.(2011). (4)	0.95(0.50–1.79)	0%	0.671

^a^ Standardized incidence rate and 95% confidence interval

^b^ Percentage of total variation attributable to statistical heterogeneity between studies (25%, low; 50%, moderate; 75%, high)

^c^ P value for heterogeneity among studies assessed with Chi-squared based Q test

^d^ Colorectal cancer

^e^ One of three cannot be merged.

### Sensitivity analysis

The results of this meta-analysis were not affected by sensitivity analysis, which were performed by excluding one study at a time. When any individual study was excluded, the corresponding pooled SIRs were not substantially altered (data not shown). The statistically similar results indicated this meta-analysis was relatively stable.

### Heterogeneity and reporting bias

In the overall cancer and NMSC groups, moderate heterogeneity between studies was observed (all P <0.10). When the study by Holle et al.[14] was deleted, the P value of heterogeneity for the overall cancer group was 0.414, indicating that this study may be the source of heterogeneity in the overall cancer group. As for the NMSC group, the P value of heterogeneity was 0.395 after removing the study by Heijl et al.[11]. The study might contribute to the heterogeneity in the the NMSC group.

The P value for Begg’s test was 0.707 (continuity corrected) and that for Egger’s test was 0.367, suggesting a low probability of publication bias.

## Discussion

To the best of our knowledge, this study is the first meta-analysis to present cancer incidence in patients with AASV. We confirmed AASV was associated with an increased risk of cancer with a SIR of 1.74 (95%CI = 1.37–2.21).

There was an moderate heterogeneity in this meta-analysis. We consider that the main source of heterogeneity was the difference in follow-up time, as the mean follow-up varied in the studies. Heijl et al.[11] reported that cumulative overall cancer incidence rates were 8% and 13% at 5 and 8 years, respectively. In addition, the study by Holle et al.[14] would play an important part in heterogeneity. When the study was excluded, the heterogeneity disappeared. An increased incidence of malignancies in AASV was not found in the study[14], which may also be partly explained by the strict use of mesna to avoid bladder cancer and by the shorter average follow-up period of 3.9 years from cohort 3 than that in other cohorts. What’s more, different AASV types and sex ratio might contribute to the heterogeneity in our study. About 65% of the samples were patients with the GPA[9,10,14], 24% were with mixed GPA/MPA[8,11], and others with Pulmonary vasculitis[12]. The reported SIRs for cancers at all sites were 1.92(95%CI = 1.31–2.71) and 1.20(95%CI = 0.71–1.89) for GPA and MPA, respectively[11]. Interestingly, men had a slightly higher relative risk of cancer than women[9,11,14]. Last, immune inhibitors and its dosage were also the source of heterogeneity. In the material presented by Heijl et al.[11], the lower CYC exposure, the lower SIR for cancers in any site. Similarly, Faurschou et al.[10] found that the risk of these malignancies was not increased for patients who never received CYC or for patients treated with cumulative CYC doses ≤ 36 g. Furthermore, Silva et al.[13] found SIR for solid malignancies of 3.92 (95% CI = 1.69–7.72) in the etanercept group among WG patients as compared to the general population, suggesting malignancy with a strong association with etanercept use.

We also observed a strongly increased risk of cancer in this paper, the risk being most pronounced for NMSC, leukemia and bladder cancer.

In regard to NMSC in this meta-analysis, a 5.18–7.73-fold increased risk is seen in AASV patients compared to the general population. The observed association between AASV and NMSC may result from the following major aspects. Firstly, most of the skin cancers occurred in sun-exposed areas, typically in the facial region[10], explained by that ultraviolet light is the main environmental cause of NMSC. Secondly, the direct causal association of AASV with NMSC may be mediated by intense immunosuppressive therapy[10]. For example, in the Westman et al.’s study[8], no case of skin cancer was observed during the first year of follow-up, however, the risk for NMSC was significantly associated with azathioprine therapy for more than 12 mouths (16-fold increase) and with glucocorticoids for more than 48 mouths (20-fold increase). Alternatively, subsequent studies in AASV were consistent with observations in the Westman et al.’s study[10,12]. Furthermore, one study found that azathioprine might hasten the induction of NMSC by sensitising the skin cell genome to ultraviolet A radiation[24]. Therefore, patients with AASV should reduce sun exposure and use sun block regularly to decrease the risk of NMSC.

With respect to leukemia risk, the results suggest an approximate 4.89 to 8.16 increased risk in patients with AASV. Some studies revealed a high risk of leukemia following treatment with CYC in AASV patients, and the risk of secondary malignancies was shown to be dependent upon the cumulative dose of CYC received[25]. Faurschou et al.[10] has demonstrated a 59-fold increased SIR of acute myeloid leukemia (AML) in patients treated with a cumulative dose of CYC > 36 g. In contrast, no case of leukemia due to milder or shorter duration of immunosuppression was found in the study by Westman et al.[8]. Besides, in the material presented by Faurschou et al.[10], the high risk of AML (SIR = 19.6, 95%CI = 4.0–57) was observed, with long latency periods of seven to sixteen years. This finding is in agreement with observations by Zycinska and colleagues, who demonstrated the risk of leukemia significantly increasing between 5 and 10 years of the follow-up observation[12]. Consequently, Patients with AASV should undergo the long-term follow-up with blood routine examination after CYC therapy to early detection of leukemia.

There is an increased SIR of bladder cancer (SIR = 3.84, 95%CI = 2.72–5.42) without heterogeneity (I^2^ = 0, P = 0.809). Our study and several studies[6,15] have demonstrated that the risk for bladder cancer was associated with the cumulative dose of CYC administered. Indeed, Hoffman et al.[6] reported a 33-fold and Talar-Williams et al.[15] a 31-fold increased risk for bladder cancer described in a cohort of WG patients followed at the NIH (National Institutes of Health, Bethesda, Maryland). The higher incidence of bladder carcinomas among the NIH patients might reflect that these patients generally received higher cumulative doses of CYC (>50 g for 64% of patients) than the patients (24% of patients) in Faurschou et al.’s study[10]. Besides, bladder cancers have been reported by another study with, like observations in hematology, a median latency period of 10.4 year after a first CYC exposure[12]. Other factors may have contributed to predict the development of bladder cancer in AASV. Microscopic non-glomerular hematuria has been recognised to be statistically significantly associated with the development of bladder cancer[15]. Although a history of smoking tobacco was not found to be an independent risk factor for the development of bladder cancer, tobacco smokers may develop bladder cancer at lower doses and earlier than non-smokers[15]. These results contribute to defining risk populations who should benefit from close and prolonged screening for bladder cancer. Moreover, oral administration of CYC was also an important predictor of increased bladder cancer risk, as a result of a French study[26]. It is also recommended that intravenous CYC should be systematically co-prescribed with mesna and hyperhydration and additional mesna prescription should be considered for oral CYC with prolonged treatment (>4–6 months)[27].

The results of our meta-analysis show no statistically significant association between AASV and kidney cancer, prostate cancer, colon cancer and breast cancer. Interestingly, kidney cancer is of note. Although its CI is wide and include the null value, either misclassification or surveillance-related bias can not be excluded, the finding may declare a carcinogenic effect of the disease process itself, as previously indicated by case reports[28]. In addition, studies of AASV reported strikingly high SIRs for cancers of the testis[8], vulva[8] and nose and ear[9], which relied on single cases and may reflect chance findings. Although not addressed in our study, the incidence of cervical neoplasia is increased in other diseases treated with immunosuppressants[29]. Thus, cervical screening is of great importance in women exposed to immunosuppressive therapies.

Several potential reasons relating to AASV that cause the increased SIRs for cancers are listed as follows. First, the used cytotoxic (predominantly CYC) therapy may increase the risk of malignancy. And in agreement of our finding, an umbrella review of 74 meta-analyses demonstrated commonly used medications including CYC may have potential increased risk of cancer[30]. Therefore, we should select a rational approach for different disease scenarios (try to avoid overuse of CYC) and focus on adverse effects of therapy[31]. Second, the dysfunction in immune system may raise the cancer risk[32]. Third, longstanding immune activation in patients with AASV may be oncogenic[33]. Fourth, vasculitis may be a paraneoplastic phenomenon, for instance, polyarteritis nodosa have been described in association with hairycell leukemia, owing to starting at the same time, concurrently improvement and the same nature[34]. Finally, some reports of cancer in association with AASV might be attributable to a coincidental association related to detection bias.

Several limitations of this meta-analysis should be acknowledged. Firstly, moderate heterogeneity was detected in the overall cancer and NMSC groups and publication bias, selection bias and a residual confounding bias may have existed although we cannot evaluate these hypotheses. Secondly, all of the studies included were partially representative as western countries with the Caucasian, and therefore, extrapolating results to other parts of the world should be interpreted cautiously. Thirdly, this meta-analysis included studies with different designs, observation period and sample size, which could introduce inherent limitations. Fourthly, due to limited data, we could not assess some confounding factors such as age and smoke. Fifthly, ANCA are now widely used as diagnostic markers for AASV, which may result in the early diagnosis of AASV, therefore it might have some bias in our final conclusions. Lastly, we could not judge whether cancer was a potential trigger or cause of AASV or a mere coincidence.

In summary, our study confirms a high incidence of cancer in the AASV population, specifically for NMSC, leukemia and bladder cancer, which might be associated with CYC use, particularly higher cumulative doses. Therefore the close follow-up is required. Periodic urine analysis, blood analysis and cervical screening should be performed. And smoking cessation and avoiding sun exposure simultaneously are recommended. Cystoscopy, bladder ultrasonography and skin biopsies should be advised in necessity. Further investigations are required to extract the accurate relations for the cancer risk to the cumulative doses. There is a continuing need for effective alternatives to toxic CYC treatment. Future studies should also focus on the underlying mechanisms between AASV and cancer risk.

## Supporting Information

S1 MOOSE Checklist MOOSE Checklist. (DOC)

S1 PRISMA Checklist PRISMA Checklist. (DOC)

## References

[1] Jennette JC, Falk RJ, Hu P Xiao H (2013) Pathogenesis of antineutrophil cytoplasmic autoantibody-associated small-vessel vasculitis. Annu Rev Pathol 8: 139–160. 10.1146/annurev-pathol-011811-132453 23347350 PMC5507606

[2] Scott DG, Watts RA (2013) Epidemiology and clinical features of systemic vasculitis. Clin Exp Nephrol 17: 607–610. 10.1007/s10157-013-0830-8 23843034

[3] Fauci AS, Haynes BF, Katz P, Wolff SM (1983) Wegener's granulomatosis: prospective clinical and therapeutic experience with 85 patients for 21 years. Ann Intern Med 98: 76–85 6336643 10.7326/0003-4819-98-1-76

[4] Flossmann O, Berden A, de Groot K, Hagen C, Harper L, Heijl C, et al (2011) Long-term patient survival in ANCA-associated vasculitis. Ann Rheum Dis 70: 488–494. 10.1136/ard.2010.137778 21109517

[5] Keogh KA, Ytterberg SR, Fervenza FC, Carlson KA, Schroeder DR, Specks U (2006) Rituximab for refractory Wegener's granulomatosis: report of a prospective, open-label pilot trial. Am J Respir Crit Care Med 173: 180–187. 10.1164/rccm.200507-1144OC 16224107 PMC2662987

[6] Hoffman GS, Kerr GS, Leavitt RY, Hallahan CW, Lebovics RS, Travis WD, et al (1992) Wegener granulomatosis: an analysis of 158 patients. Ann Intern Med 116: 488–498 1739240 10.7326/0003-4819-116-6-488

[7] Berden A, Goceroglu A, Jayne D, Luqmani R, Rasmussen N, Bruijn JA, et al (2012) Diagnosis and management of ANCA associated vasculitis. Bmj 344: e26 10.1136/bmj.e26 22250224

[8] Westman KW, Bygren PG, Olsson H, Ranstam J, Wieslander J (1998) Relapse rate, renal survival, and cancer morbidity in patients with Wegener's granulomatosis or microscopic polyangiitis with renal involvement. J Am Soc Nephrol 9: 842–852 9596082 10.1681/ASN.V95842

[9] Knight A, Askling J, Ekbom A (2002) Cancer incidence in a population-based cohort of patients with Wegener's granulomatosis. Int J Cancer 100: 82–85. 10.1002/ijc.10444 12115591

[10] Faurschou M, Sorensen IJ, Mellemkjaer L, Loft AG, Thomsen BS, Tvede N, et al (2008) Malignancies in Wegener's granulomatosis: incidence and relation to cyclophosphamide therapy in a cohort of 293 patients. J Rheumatol 35: 100–105 17937462

[11] Heijl C, Harper L, Flossmann O, Stucker I, Scott DG, Watts RA, et al (2011) Incidence of malignancy in patients treated for antineutrophil cytoplasm antibody-associated vasculitis: follow-up data from European Vasculitis Study Group clinical trials. Ann Rheum Dis 70: 1415–1421. 10.1136/ard.2010.145250 21616914

[12] Zycinska K, Kostrzewa-Janicka J, Nitsch-Osuch A, Wardyn K (2013) Cancer incidence in pulmonary vasculitis. Adv Exp Med Biol 788: 349–353. 10.1007/978-94-007-6627-3_47 23835997

[13] Silva F, Seo P, Schroeder DR, Stone JH, Merkel PA, Hoffman GS, et al (2011) Solid malignancies among etanercept-treated patients with granulomatosis with polyangiitis (Wegener's): long-term followup of a multicenter longitudinal cohort. Arthritis Rheum 63: 2495–2503. 10.1002/art.30394 21484770 PMC3149780

[14] Holle JU, Gross WL, Latza U, Nolle B, Ambrosch P, Heller M, et al (2011) Improved outcome in 445 patients with Wegener's granulomatosis in a German vasculitis center over four decades. Arthritis Rheum 63: 257–266. 10.1002/art.27763 20862686

[15] Talar-Williams C, Hijazi YM, Walther MM, Linehan WM, Hallahan CW, Lubensky I, et al (1996) Cyclophosphamide-induced cystitis and bladder cancer in patients with Wegener granulomatosis. Ann Intern Med 124: 477–484 8602705 10.7326/0003-4819-124-5-199603010-00003

[16] Mahr A, Heijl C, Le Guenno G, Faurschou M (2013) ANCA-associated vasculitis and malignancy: current evidence for cause and consequence relationships. Best Pract Res Clin Rheumatol 27: 45–56. 10.1016/j.berh.2012.12.003 23507056

[17] Stroup DF, Berlin JA, Morton SC, Olkin I, Williamson GD, Rennie D, et al (2000) Meta-analysis of observational studies in epidemiology: a proposal for reporting. Meta-analysis Of Observational Studies in Epidemiology (MOOSE) group. Jama 283: 2008–2012 10789670 10.1001/jama.283.15.2008

[18] Stang A (2010) Critical evaluation of the Newcastle-Ottawa scale for the assessment of the quality of nonrandomized studies in meta-analyses. Eur J Epidemiol 25: 603–605. 10.1007/s10654-010-9491-z 20652370

[19] Mantel N, Haenszel W (1959) Statistical aspects of the analysis of data from retrospective studies of disease. J Natl Cancer Inst 22: 719–748 13655060

[20] DerSimonian R, Laird N (1986) Meta-analysis in clinical trials. Control Clin Trials 7: 177–188 3802833 10.1016/0197-2456(86)90046-2

[21] Higgins JP, Thompson SG (2002) Quantifying heterogeneity in a meta-analysis. Stat Med 21: 1539–1558. 10.1002/sim.1186 12111919

[22] Begg CB, Mazumdar M (1994) Operating characteristics of a rank correlation test for publication bias. Biometrics 50: 1088–1101 7786990

[23] Egger M, Davey Smith G, Schneider M, Minder C (1997) Bias in meta-analysis detected by a simple, graphical test. Bmj 315: 629–634 9310563 10.1136/bmj.315.7109.629 PMC2127453

[24] O'Donovan P, Perrett CM, Zhang X, Montaner B, Xu YZ, Harwood CA, et al (2005) Azathioprine and UVA light generate mutagenic oxidative DNA damage. Science 309: 1871–1874. 10.1126/science.1114233 16166520 PMC2426755

[25] Pedersen-Bjergaard J, Specht L, Larsen SO, Ersboll J, Struck J, Hansen MM, et al (1987) Risk of therapy-related leukaemia and preleukaemia after Hodgkin's disease. Relation to age, cumulative dose of alkylating agents, and time from chemotherapy. Lancet 2: 83–88 2885581 10.1016/s0140-6736(87)92744-9

[26] Le Guenno G, Mahr A, Pagnoux C, Dhote R, Guillevin L (2011) Incidence and predictors of urotoxic adverse events in cyclophosphamide-treated patients with systemic necrotizing vasculitides. Arthritis Rheum 63: 1435–1445. 10.1002/art.30296 21337321

[27] Monach PA, Arnold LM, Merkel PA (2010) Incidence and prevention of bladder toxicity from cyclophosphamide in the treatment of rheumatic diseases: a data-driven review. Arthritis Rheum 62: 9–21. 10.1002/art.25061 20039416

[28] Roussou M, Dimopoulos SK, Dimopoulos MA, Anastasiou-Nana MI (2008) Wegener's granulomatosis presenting as a renal mass. Urology 71: 547.e541–542. 10.1016/j.urology.2007.11.046 18342212

[29] Nath R, Mant C, Luxton J, Hughes G, Raju KS, Shepherd P, et al (2007) High risk of human papillomavirus type 16 infections and of development of cervical squamous intraepithelial lesions in systemic lupus erythematosus patients. Arthritis Rheum 57: 619–625. 10.1002/art.22667 17471531

[30] Ioannidis JP, Zhou Y, Chang CQ, Schully SD, Khoury MJ, Freedman AN (2014) Potential increased risk of cancer from commonly used medications: an umbrella review of meta-analyses. Ann Oncol 25: 16–23. 10.1093/annonc/mdt372 24310915 PMC3868319

[31] Bosch X, Guilabert A, Espinosa G, Mirapeix E (2007) Treatment of antineutrophil cytoplasmic antibody associated vasculitis: a systematic review. Jama 298: 655–669. 10.1001/jama.298.6.655 17684188

[32] Weyand CM, Goronzy JJ, Kurtin PJ (2006) Lymphoma in rheumatoid arthritis: an immune system set up for failure. Arthritis Rheum 54: 685–689. 10.1002/art.21674 16508924

[33] Zintzaras E, Voulgarelis M, Moutsopoulos HM (2005) The risk of lymphoma development in autoimmune diseases: a meta-analysis. Arch Intern Med 165: 2337–2344. 10.1001/archinte.165.20.2337 16287762

[34] Fortin PR (1996) Vasculitides associated with malignancy. Curr Opin Rheumatol 8: 30–33 8867536 10.1097/00002281-199601000-00005

