# Supplementary material for: Incidence of Cancer in ANCA-Associated Vasculitis: A Meta-Analysis of Observational Studies
Source: PLoS One. 2015 May 14;10(5):e0126016. doi: 10.1371/journal.pone.0126016 (PMC4431871; doi:10.1371/journal.pone.0126016)
Supplement: S1 MOOSE Checklist — (DOC) [file pone.0126016.s001.doc]

**Meta-analysis of Observational Studies in Epidemiology (MOOSE) Checklist**

**Incidence of Cancer in ANCA-associated vasculitis: A Meta-Analysis of Observational Studies**

| **Criteria** | | **Brief description of how the criteria were handled in the meta-analysis** |
| --- | --- | --- |
| **Reporting of background should include** | |  |
| √ | Problem definition | A growing number of studies suggest that AASV is related to an increased risk of cancer, but the risk of overall cancer appeared to be somewhat reduced in one study, Moreover, cancer type-specific analyses demonstrated an significantly increased risk of non-melanoma skin cancer,leukemia and bladder cancer in some studies. However, the reported risk is different. Given the fact that individual studies may have insufficient statistical power because of sample size, therefore, we undertook the present meta-analysis to quantitatively confirm the incidence of cancer in AASV patients versus the general population, which may provide a realistic perspective on risk in the clinical setting. |
| √ | Hypothesis statement | AASV patients treatment with cyclophosphamide (CYC) are at increased risk of late-occurring malignancies |
| √ | Description of study outcomes | SIR of cancer |
| √ | Type of exposure or intervention used | AASV as one of the exposure interests |
| √ | Type of study designs used | cohort studies that estimating the influence of AASV on cancer risk with SIR and their 95%CI of overall cancer. |
| √ | Study population | AASV patients versus the general population |
| **Reporting of search strategy should include** | |  |
| √ | Qualifications of searchers | The two experienced investigators (SG and WS ) are indicated in the authors list |
| √ | Search strategy, including time period included in the synthesis and keywords | A PubMed and EMBASE databases were searched for all articles published before September 1st, 2014. Keywords: “cancer and ANCA-associated vasculitis” |
| √ | Databases and registries searched | PubMed and EMBASE |
| √ | Search software used, name and version, including special features | We did not employ a search software. Endnote was used  to merge retrieved citations |
| √ | Use of hand searching | We hand-searched references of retrieved papers for  additional references |
| √ | List of citations located and those excluded, including justifications | Figure 1 |
| √ | Method of addressing articles published in languages other than English | We placed no restrictions on language; We were able to obtained all articles potentially eligible for inclusion in English language |
| √ | Method of handling abstracts and unpublished studies | We did not include unpublished or abstract only  publications |
| √ | Description of any contact with authors | When needed, we contacted the original anthor for clarification |
| **Reporting of methods should include** | |  |
| √ | Description of relevance or appropriateness of studies assembled for assessing the hypothesis to be tested | Detailed inclusion and exclusion criteria are described in the paper |
| √ | Rationale for the selection and coding of data | The following data were extracted : the authors, publication year, country, study design, AASV phenotypes studied, period of follow-up, mean/median observation period, cumulative observation period, number of patients studied, number of cancers observed in the cohort, patients’gender and age, and SIR with its 95% CI, gender of cancer patients. |
| √ | Assessment of confounding | We conducted a subgroup analysis to stratify for site-specific cancers. |
| √ | Assessment of study quality, including blinding of quality assessors; stratification or regression on possible predictors of study results | We used a modified version of the Newcastle Ottawa Scale (NOS) to assess the quality of each study. |
| √ | Assessment of heterogeneity | We used the P and I^2^ value to assess heterogeneity |
| √ | Description of statistical methods in sufficient detail to be replicated | We mentioned type of analysis we used (meta-analysis and subgroup meta-analysis) and type of software we used Stata 10.0 (College Station, TX, USA) |
| √ | Provision of appropriate tables and graphics | Table1 showing characteristics of included studies, Table 2 showing results of Quality assessment, Table 3 showing Results of Subgroup analysis, Figure 1 showing literature search flow diagra, Figure 2 showing forest plot |
| **Reporting of results should include** | |  |
| √ | Graph summarizing individual study estimates and overall estimate | Figure 2 |
| √ | Table giving descriptive information for each study included | Table 1 |
| √ | Results of sensitivity testing | Table 3 |
| √ | Indication of statistical uncertainty of findings | SIR, 95% CI and I^2^ |
| **Reporting of discussion should include** | |  |
| √ | Quantitative assessment of bias | Results of Funnel plot and subgroup analyses is discussed |
| √ | Justification for exclusion | See above |
| √ | Assessment of quality of included studies | Table 2 |
| **Reporting of conclusions should include** | |  |
| √ | Consideration of alternative explanations for observed results | Firstly, moderate heterogeneity was detected in the overall cancer and NMSC groups and publication bias and a residual confounding bias may have existed although we cannot assess these hypotheses. Secondly, all of the studies included were partially representative as western countries with the Caucasian, and therefore, extrapolating results to other parts of the world should be interpreted cautiously. Thirdly, this meta-analysis included studies with different designs, observation period and sample size, which could introduce inherent limitations. Fourthly, we could not evaluate some confounding factors such as age and environment triggers because of limited data. Lastly, we could not judge whether cancer was a potential trigger or cause of AASV or a mere coincidence. |
| √ | Generalization of the conclusions | Our study confirm a high incidence of cancer in the AASV population, specifically for NMSC, leukemia and bladder cancer. This risk appears to be associated with CYC use, particularly higher cumulative doses. |
| √ | Guidelines for future research | More studies are needed to determine whether reducing exposure to immunosuppressive agents can reduce the cancer risk. There is a continuing need for effective alternatives to toxic CYC treatment. Future studies should also focus on the underlying mechanisms between AASV and cancer risk. |
| √ | Disclosure of funding source | This work was supported by the National Nature Science Foundation of China (NSFC) (No. 81200531 for Shuwang Ge; No. 81470948 and 81270770 for Gang Xu). |
